# Healthcare Costs and Resource Use Associated With Cervical Intraepithelial Neoplasia and Cervical Conization: A Retrospective Study of German Statutory Health Insurance Claims Data

**DOI:** 10.36469/001c.35329

**Published:** 2022-05-26

**Authors:** Anna-Janina Stephan, Miriam Reuschenbach, Kunal Saxena, Vimalanand S. Prabhu, Christian Jacob, Kim M. Schneider, Wolfgang Greiner, Regine Wölle, Monika Hampl

**Affiliations:** 1 Department of Market Access MSD Sharp & Dohme GmbH; 2 Global Medical and Scientific Affairs MSD Sharp & Dohme GmbH; 3 Center for Observational and Real-World Evidence (CORE) Merck & Co., Inc.; 4 EU Real World Evidence Xcenda GmbH; 5 Department of Health Economics and Health Care Management, Bielefeld School of Public Health Bielefeld University; 6 Department of Gynecology University of Duesseldorf

**Keywords:** healthcare costs, cervical conization, human papilloma virus (HPV), cervical intraepithelial neoplasia (CIN), claims data, Germany

## Abstract

**Background:** Cervical intraepithelial neoplasia (CIN) can be a consequence of human papillomavirus (HPV) infection. High-grade CIN (CIN2/CIN3) may develop from persistent HPV infection and progress to cervical cancer if left untreated. Management of CIN includes conservative surveillance or ablation and excision by conization. Internationally, CIN and its treatment generate a considerable economic burden, but no current data regarding costs and resource use from the perspective of the German statutory health insurance exist.

**Objectives:** The aim of this study was to explore the health economic burden in women with CIN diagnoses who either underwent cervical conization or were managed conservatively.

**Methods:** We conducted a retrospective claims data analysis using the InGef Research Database from 2013 to 2018. Healthcare costs and resource utilization in a 24-month observation period (1:1:1 matching) were compared in 18- to 45-year-old women with CIN (1-3) who underwent a conization procedure (study cohort 1) and in women with CIN (1-3) who did not undergo conization (study cohort 2) to women with neither CIN nor conization (control group).

**Results:** For each group, 2749 women were identified. Mean total healthcare costs after 24 months were higher in study cohort 1 (€4446, *P*<.01) and study cohort 2 (€3754, *P*=.09) compared with the control group (€3426). Comparing study cohort 1 and 2 to controls, mean differences were highest in age groups 41-45 years (cohort 1: €5115 vs €3354, *P*<.01; cohort 2: €4152 vs €3354, *P*=.14). Significantly more women were hospitalized at least once in study cohort 1 (57.46%, *P*<.01) and study cohort 2 (38.74%, *P*<.01) compared with the control group (31.14%). Frequency of outpatient physician visits was significantly higher in both study cohorts (43.23 visits, P<.01 and 38.60 visits, *P*<.01) compared with the control group (32.07 visits).

**Conclusion:** Our results revealed 30% and 10% increased total healthcare costs in women with CIN undergoing invasive treatment (study cohort 1) and conservative management (study cohort 2), respectively, compared with a control group of women with no CIN in a 2-year follow-up period.

## BACKGROUND

Cervical intraepithelial neoplasia (CIN) can be a consequence of human papillomavirus (HPV) infection. Oncogenic HPV infection of the uterine cervix is very common and can be acquired throughout life.^1^ In Germany, a prevalence of 23% in 25- to 26-year-old women was reported for 2009-2010.^2^ About 90% of cervical HPV infections clear naturally within a period of few months or years.^3,4^ High-grade CIN (CIN2 and CIN3), which are considered precancerous lesions, develop in about 5% of women with persistent infection over a period of 1-3 years.^5,6^ If left untreated, high-grade CIN may progress to cervical cancer.^7^

Primary prevention through HPV vaccination, secondary prevention through screening for precancerous lesions, and treatment of CIN (usually through cervical conization) are funded by Germany’s statutory health insurance (SHI). HPV vaccination was introduced in Germany in 2007 and recommended with mandatory funding for 12- to 17-year-old girls until 2014.^8^ In 2014, the recommended vaccination age was updated to 9-14 years including catch-up vaccination until the age of 17 years.^9^ In 2018, this recommendation was extended to boys.^10^ From 1971 until 2019, an annual opportunistic Pap test screening was funded by the SHI for women aged 20 years and older.^11^ HPV tests were mainly used if the Pap test was equivocal or if it was paid out of pocket. Since January 1, 2020, a new organized screening program comprises annual screening for women aged 20-34 years using Pap test and cotesting (Pap test + HPV test) in 3-year intervals for women 35 years and older.^12,13^

Women continue to present with HPV-associated cervical lesions in Germany. Estimates from 2011-2013^14^ and 2016^15^ suggest that about 50 000 women underwent histological diagnostic examination following positive or equivocal cytology results per year, with resulting CIN diagnoses in most cases. For 2013-2015, a 3-year CIN3 prevalence of 0.3% was estimated for women born in 1990.^16^ Other sources reported 4640 new cervical cancer cases for 2012, projecting 4300 cases for 2016.^17^

The German guideline for prevention of cervical cancer^18^ recommends watching and re-evaluating histopathologically verified CIN1 and CIN2 after 6 months. Especially in women under 25 years of age or during pregnancy, a conservative approach with cytologic and colposcopic surveillance is recommended. After persistence over 2 years, both further careful observation and surgical interventions (ablation or excision) can be considered for CIN1, whereas persisting CIN2 lesions should be surgically removed. For CIN3, removal of the abnormal tissue, eg, by conization, is generally recommended except for women under 25 years of age, where a conservative approach including close re-evaluations via colposcopy, cytology, and HPV tests can also be conducted. Cervical conization is the standard surgical procedure used for diagnosis and treatment of high-grade CIN. Conizations can be done with either scalpel, laser, or an electrosurgical instrument typically referred to as LEEP (loop electrosurgical excision procedure).^19^ For Germany, estimates on frequency of annual conizations vary considerably between 50 000 and 140 000 (based on 2006-2009 extrapolated estimates).^18,20,21^

Besides the clinical burden, CIN also has a health economic impact on the healthcare system. For Germany, estimated average costs of managing and treating precancerous cervical lesions 12 months from diagnosis based on data for 138 women ranged from €943 to €3174 in 2004.^22^ However, analyses of larger samples providing more recent evidence on the healthcare resource utilization (HRU) and costs of women with CIN are currently lacking. This applies to women with CIN who undergo surgical treatment and to those with CIN undergoing conservative disease management.

The aim of this study was to explore the health economic burden of CIN, in terms of healthcare costs and HRU, in women who either underwent surgical treatment (cervical conization) or were managed conservatively, compared with women without CIN diagnoses and without conizations in a 2-year time frame from the perspective of the German SHI using 2013-2018 claims data.

## METHODS

### Study Design

We conducted a retrospective matched cohort claims data analysis to estimate the health economic burden attributable to CIN diagnoses treated with surgery or managed conservatively, in terms of healthcare costs and HRU from the perspective of the German SHI. Healthcare costs and HRU in women with CIN who underwent a conization procedure (study cohort 1) and women with CIN who did not undergo conization (study cohort 2) were compared with women with neither a CIN diagnosis nor a conization (control group) during a 24-month observation period.

### Data Source

The Institute for Applied Health Research Berlin (InGef) database consists of about 9 million covered lives and includes the healthcare claims data (HRU and costs of services) from about 60 different health insurances (covering over half the overall number of health insurances in Germany) in an anonymized case-by-case format. The age- and sex-adjusted analysis sample of the InGef Research Database includes approximately 4 million covered lives structured to represent the respective German population demographics (structure of age and gender according to the Federal Office of Statistics). This InGef Research Database comprises a geographically well-distributed 4.8% of the German population^23^ and 5.5% of the German SHI population^24^ and has demonstrated good external validity to the German population in terms of morbidity, mortality, and drug use.^25^ Details on included data domains in the InGef Research Database are available in the **Supplement,** Section 1.

### Data Protection

The analysis of German SHI claims data is permitted by social law, and no review by an independent ethics committee or institutional review board was required to conduct this study.

Claims data from the participating SHIs are joined in a specialized trust center, anonymized, and subsequently transferred to InGef. As the raw dataset is not allowed to leave the secured storage facilities, all analyses were conducted by an InGef analyst in accordance with a prespecified study protocol.

### Study Population

**Figure 1.**
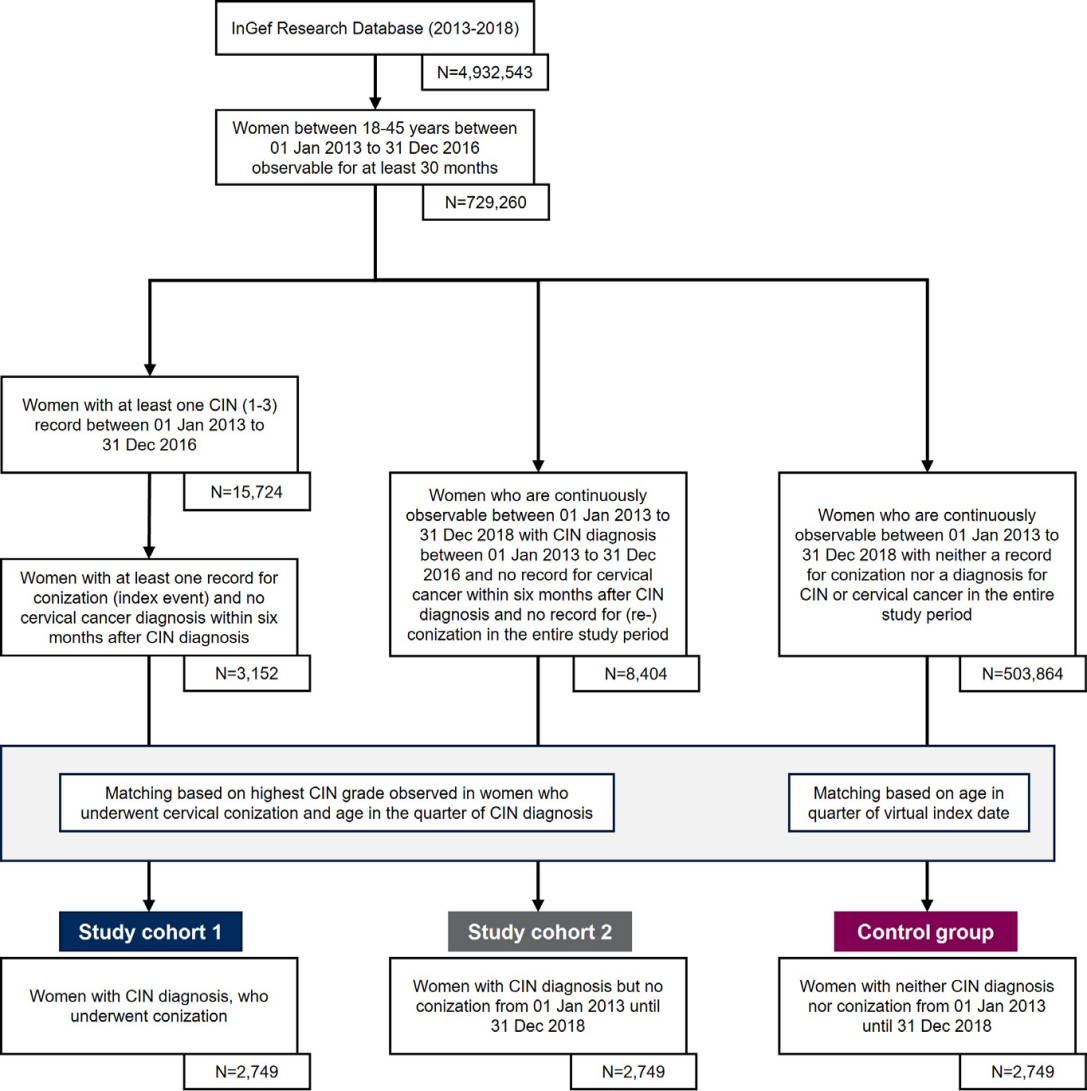
Flow Chart for Selection of Study Groups and Controls Abbreviations: CIN, cervical intraepithelial neoplasia; InGef, Institute for Applied Health Research.

The overall study population was drawn from the InGef Research Database using data from 2013-2018 for all women aged 18-45 years between 2013 and 2016 (Figure 1).

**Study population eligible for study cohort 1: CIN patients undergoing surgical treatment (conization):** From the overall study population (18- to 45-year-old women in 2013-2016), all women were identified if they had at least 1 diagnosis for CIN1/2/3 (identified by ICD-10-GM codes) in the time frame from January 1, 2013, until December 31, 2016. Therefrom, all women with a medical record for conization (index event) within 6 months after a CIN diagnosis in the time frame between July 1, 2013, and December 31, 2016, were identified. The identified women needed to be continuously observable for at least 24 months beginning with the index event and 6 months prior to conization (baseline period) to assess baseline demographics and baseline clinical characteristics including a record of the most severe CIN diagnosis. In addition, no record for conization, reconization, or cervical cancer (ICD-10-GM code C53.-) in the 6-month baseline period was allowed.

**Study population eligible for study cohort 2: CIN patients with conservative management**: From the overall study population (18- to 45-year-old women in 2013-2016), all women in the InGef Research Database were identified if they had at least 1 diagnosis for CIN1/2/3 in the time frame from January 1, 2013, until December 31, 2016. The identified women in study cohort 2 were included only if they did not have any record for cervical conization or reconization or cervical cancer (ICD-10-GM code C53.-) in the complete study period (January 1, 2013–December 31, 2018). Therefore, study cohort 2 needed to be continuously observable for the complete study period (January 1, 2013–December 31, 2018).

**Study population eligible for control group: Women with neither CIN diagnoses nor conization:** From the overall study population (18- to 45-year-old women in 2013-2016), all women in the InGef Research Database were identified if they neither had a diagnosis for CIN1/2/3, cervical cancer (ICD-10-GM code C53.-) nor a medical record for cervical conization in the complete study period (January 1, 2013–December 31, 2018). Therefore, the identified women needed to be continuously observable for the entire study period from January 1, 2013, to December 31, 2018.

### Matching

For the comparison of HRU and costs between patients with CIN and conization (study cohort 1) and women with CIN who did not undergo a conization (study cohort 2) vs women without both CIN diagnoses and conization (control group), a 1:1:1 direct, exact matching without replacement was performed. Study cohort 2 was matched on age and the most severe CIN grade in the same year-specific quarter to study cohort 1. The control group was matched on age in the respective calendar year to both study cohorts. To define a common follow-up period for all 3 study groups, the date of the index event of study cohort 1 (conization after CIN diagnosis) was assigned to both study cohort 2 and the control group as a virtual index event to mark the start of the 24-month follow-up period.

### Outcomes

Healthcare costs during the 24-month follow-up period (starting with the index conization/virtual index date) were analyzed as all-cause costs for outpatient care, inpatient care, outpatient pharmaceuticals, aids and remedies, and sick leave costs, and summarized as total costs. As information on sick leave costs and aids and remedies costs were not complete for all individuals in the InGef Research Database, missing data were imputed by using the mean sick leave costs or aids and remedies costs of women with available sick leave data or aids and remedies data, respectively. Valid sick leave data were available for 77.48% of women in study cohort 1, 76.97% of women in study cohort 2, and 94.94% of women in the control group. Valid aids and remedies data were available for 79.92%, 79.92%, and 79.99%, respectively.

HRU was analyzed in terms of inpatient and outpatient care. Inpatient care included proportion of women with all- cause hospitalizations, frequency of all-cause hospitalizations, and length of hospital stay. Outpatient care included number and proportion of women with all-cause outpatient visits, and frequency of all-cause outpatient physician visits.

Baseline demographics and clinical characteristics were assessed during the 6 months before the index conization in study cohort 1 (virtual index event for study cohort 2 and control group) including geographic region, living place, insurance member type, highest CIN diagnosis, and Charlson Comorbidity Index (CCI) score.^26^ For further details on the identification of baseline demographics and clinical characteristics, eg, codes used for identification, see **Supplement, Section 2.**

### Exposures

**Cervical conization:** The index cervical conization was identified using the German classification of operation and procedures (OPS) codes for conization (no reconization, see **Supplement, Section 3, Table 2**).

**CIN diagnoses:** For the identification of CIN we used the *International Statistical Classification of Diseases, 10th Revision, German Modification*) (ICD-10-GM) codes. Women with at least 1 “verified” outpatient diagnosis or primary or secondary inpatient diagnosis in the respective period were considered as having CIN. The following ICD-10-GM codes were used for the identification of patients with CIN diagnoses: N87.0 (CIN1), N87.1 (CIN2), N87.2 and/or D06 (CIN3).

**Statistical methods:** Healthcare utilization and costs were quantified in all 3 groups. Mean, SD, minimum, 25th percentile, median, 75th percentile, and maximum were presented for all patients during the study period. Differences between each of the groups were tested for statistical significance using χ^2^ tests and Fisher’s exact test as applicable for categorical variables and *t* tests for continuous variables. Statistical significance was defined as *P<*.05. As a sensitivity analysis, we accounted for outliers in the cost data through winsorization. Costs were winsorized at the 98th percentiles by assigning costs of the 98th percentile to costs above that value. Analyses were performed with Microsoft R Open version 3.5.0.

## RESULTS

### Patient Selection

There were 729 260 women aged 18-45 years in the InGef Research Database between July 1, 2013, and December 31, 2016. After applying the inclusion criteria, 3152 women were eligible for study cohort 1. There were 8404 women eligible for study cohort 2 and 503 864 for the control group. After matching, 2749 women were considered for each of the 3 groups, with a mean age of 33.6 years (Table 1). The mean age of eligible women before the matching in study cohort 1 (33.5 years) and study cohort 2 (33.0 years) slightly increased after the matching. Even though geographic region, living place, and insurance membership status were not used as matching criteria, all 3 groups showed similar distributions in these categories after matching.

**Table 1.** Baseline Demographic and Clinical Characteristics During the 6-Month Baseline Period Before Index Date Before and After Matching

	**Study Cohort 1**				**Study Cohort 2**				**Control Group**	
	**Before Matching**		**After Matching**		**Before Matching**		**After Matching**		**After Matching**	
	**n**	**%**	**n**	**%**	**n**	**%**	**n**	**%**	**n**	**%**
Total 18- to 45-year-old women	3152	100.00	2749	100.00	8404	100.00	2749	100.00	2749	100.00
Mean age (SD)	33.53 (5.97)		33.60 (6.11)		32.95 (7.33)		33.60 (6.11)		33.60 (6.11)	
Age groups (years)										
18-19	<5	—	<5	—	187	2.23	<5	—	<5	—
20-26	377	11.96	345	12.55	1695	20.17	345	12.55	345	12.55
27-30	690	21.89	598	21.75	1461	17.38	598	21.75	598	21.75
31-35	929	29.47	764	27.79	1742	20.73	764	27.79	764	27.79
36-40	657	20.84	577	20.99	1647	19.60	577	20.99	577	20.99
41-45	495	15.70	461	16.77	1672	19.90	461	16.77	461	16.77
Year of index conization										
2013a	395	12.53	338	12.30	—	—	—	—	—	—
2014	914	29.00	798	29.03	—	—	—	—	—	—
2015	904	28.68	767	27.90	—	—	—	—	—	—
2016	939	29.79	846	30.77	—	—	—	—	—	—
Geographic region										
East	225	7.14	200	7.28	572	6.81	196	7.13	162	5.89
North	664	21.07	568	20.66	1856	22.08	605	22.01	503	18.30
South	964	30.58	837	30.45	2693	32.04	843	30.67	990	36.01
West	1285	40.77	1132	41.18	3245	38.61	1098	39.94	1085	39.47
Unknown	14	0.44	12	0.44	38	0.45	7	0.25	9	0.33
Residence										
Rural	836	26.52	730	26.56	2248	26.75	721	26.23	750	27.28
Urban	2316	73.48	2019	73.44	6153	73.22	2027	73.74	1999	72.72
Unknown	0	0.00	0	0.00	<5	—	<5	—	0	0.00
Insurance member status										
Member	2880	91.37	2513	91.42	7337	87.30	2459	89.45	2347	85.38
Dependent coverage	246	7.80	212	7.71	1004	11.95	270	9.82	383	13.93
Retired	26	0.82	24	0.87	63	0.75	20	0.73	18	0.65
Unknown	0	0.00	0	0.00	0	0.00	<5	—	<5	—
Highest CIN diagnosis										
CIN1	118	3.74	116	4.22	2919	34.73	116	4.22	—	—
CIN2	414	13.13	372	13.53	1622	19.30	372	13.53	—	—
CIN3	2620	83.12	2261	82.25	3863	45.97	2261	82.25	—	—
CCI group										
0	1557	49.40	356	49.33	4472	53.21	1428	51.95	1554	56.53
1	1022	32.42	878	31.94	2849	33.90	940	34.19	871	31.68
2	255	8.09	230	8.37	516	6.14	185	6.73	165	6.00
3	204	6.47	181	6.58	371	4.41	124	4.51	102	3.71
4+	114	3.62	104	3.78	196	2.33	72	2.62	57	2.07

Abbreviations: CCI, Charlson Comorbidity Index; CIN, cervical intraepithelial neoplasia; HPV, human papillomavirus; North: Schleswig-Holstein, Hamburg, Bremen, Lower Saxony, Mecklenburg-Western Pomerania; East: Thuringia, Brandenburg, Berlin, Saxony, Saxony-Anhalt; West: North Rhine, Saarland, Rhineland-Palatinate, Hesse; South: Bavaria, Baden-Württemberg.

Study cohort 1: women with CIN diagnoses and a subsequent cervical conization; study cohort 2: women with CIN diagnoses and no cervical conization in the entire study period; control group: women with neither a CIN diagnosis nor a conization in the entire study period.

^a^Due to the required baseline period of 6 months before the index conization, for 2013, only index conizations between July and December could be considered. For consecutive years, conizations from January to December could be included.

Before matching, in 83.1% of women eligible for study cohort 1, CIN3 was the highest documented diagnosis in the 6 months before a conization. Among women eligible for study cohort 2, CIN3 was documented in 46.0% and CIN1 in 34.7% as the highest documented diagnosis (Table 1). After matching, the distribution of highest documented CIN grades barely changed in study cohort 1, with 82.2% of women with CIN3. In study cohort 2, however, the distribution of highest CIN diagnoses changed accordingly to the distribution of CIN grades in study cohort 1, with 82.2% of women with a CIN3 diagnosis and 4.2% women with a CIN1 diagnosis.

Before matching, mean CCI in women eligible for study cohort 1 and study cohort 2 was 0.77 and 0.72, respectively. After matching, mean CCI increased in both study cohorts with highest mean CCI among women in study cohort 1 (0.90), followed by study cohort 2 (0.75), and the control group (0.65).

### Healthcare Costs During 24-Month Follow-up

**Figure 2.**
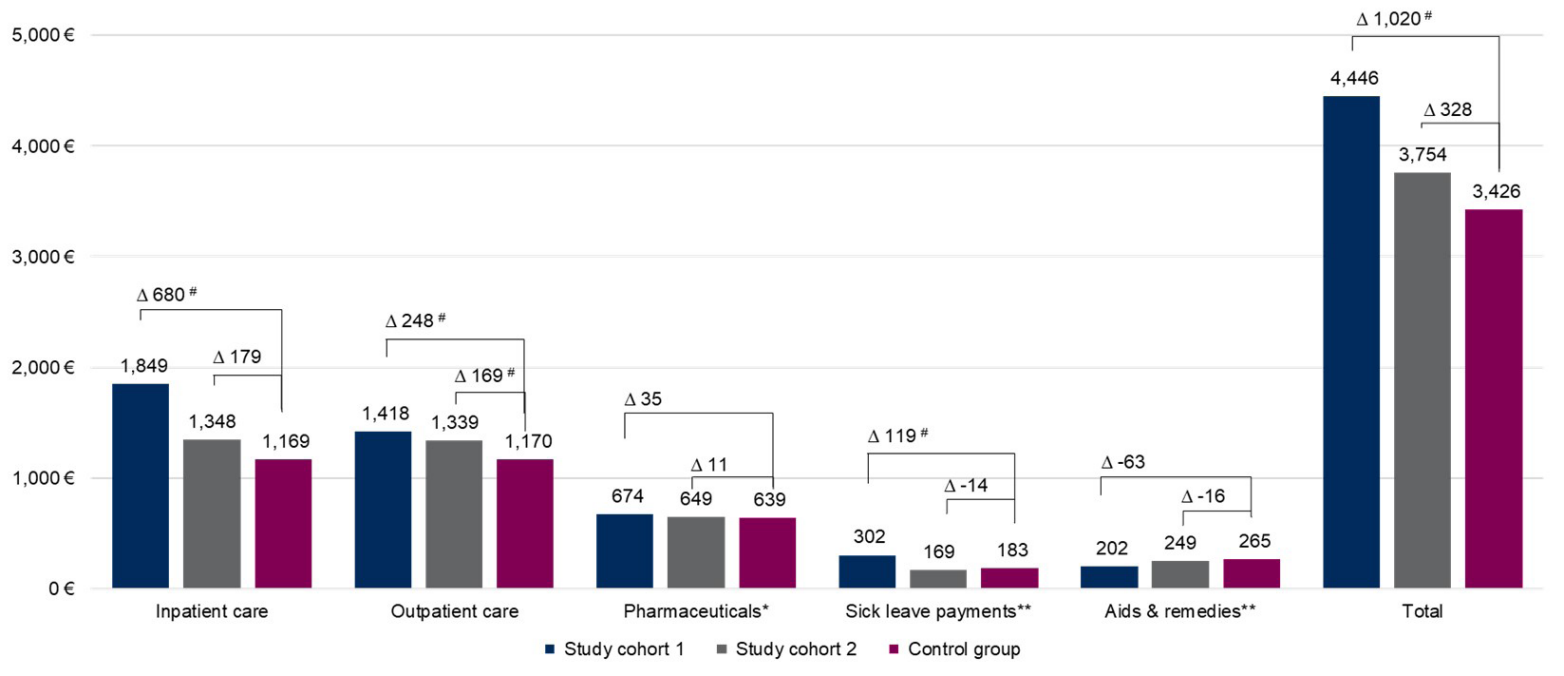
Mean All-Cause Healthcare Costs During 24-Month Follow-up in Women 18-45 ∆, Mean difference. Costs are presented in euros (€). Study cohort 1: women with CIN diagnoses and a subsequent cervical conization; study cohort 2: women with CIN diagnoses and no cervical conization in the entire study period; control group: women with neither a CIN diagnosis nor a conization in the entire study period. # Significant differences according to *t* test (*P*<.05). *Including costs for outpatient pharmaceuticals only. Costs for inpatient applied pharmaceuticals are included in costs for inpatient care. **Including imputed sick leave costs and aids and remedies costs for missing data.

Within 24 months of follow-up, mean total healthcare costs in women with CIN and conization (€4446) as well as in women with CIN and no conization (€3754) were €1020 (*P<*.01) and €328 (*P=*.09) higher compared with the control group (€3426), respectively (Table 2). This corresponds to 30% and 10% increased total healthcare costs in women with CIN undergoing invasive treatment (study cohort 1) and conservative management (study cohort 2), respectively, compared with the control group. In both study groups, costs were mainly driven by inpatient and outpatient care, with inpatient care being the main cost driver for women in study cohort 1. In study cohort 2, mean costs for inpatient and outpatient care were almost equal (Figure 2). In both study cohorts, the highest mean total healthcare costs were observed in the age group 41- 45 years (€5115, *P<*.01 and €4152 vs €3354, *P=*.14) (Table 2). The highest mean total cost differences between study cohort 1 and controls were observed in the 41-45 (€5115 vs €3354, *P<*.01) and 36-40 (€4846 vs €3317, *P<*.01) age groups. In study cohort 2, highest mean differences were observed for the 41-45 (€4152 vs €3354, *P=*.14) and 20-26 years (€3073 vs 2605, *P=*.31) age groups. In study cohort 1, mean total cost difference in the 20-26 age group was also statistically significant (€3651 vs €2605, *P=*.02).

**Table 2.** All-Cause Healthcare Costs During 24-Month Follow-up in Women 18-45, Stratified by Age and Cost Domains

	**Costs (€)**																					***P* Value^a^**	
	**Study Cohort 1**							**Study Cohort 2**							**Control Group**							**Study Cohort 1**	**Study Cohort 2**
	**Mean**	**SD**	**Min**	**Q1**	**Med**	**Q3**	**Max**	**Mean**	**SD**	**Min**	**Q1**	**Med**	**Q3**	**Max**	**Mean**	**SD**	**Min**	**Q1**	**Med**	**Q3**	**Max**		
Total costs																							
Total (18-45 y)	4446	7115	74	1297	2368	5107	85 866	3754	7004	0	851	1677	4292	106 470	3426	7490	0	591	1238	3755	113 381	<.01	.09
18-19 y	—	—	—	—	—	—	—	—	—	—	—	—	—	—	—	—	—	—	—	—	—	—	—
20-26 y	3651	6199	280	1233	1967	3998	83 316	3073	6732	82	745	1320	3011	80 952	2605	5283	0	588	1065	2819	54 463	.02	.31
27-30 y	3987	5448	128	1204	2207	5160	55 948	3751	6198	39	852	1792	4489	60 327	3432	6249	0	639	1429	4333	67 299	.10	.38
31-35 y	4468	6346	74	1411	2453	5264	58 163	3913	6446	52	894	1908	4901	80 940	3930	9007	0	610	1336	4480	113 381	.18	.97
36-40 y	4846	9053	113	1249	2338	4927	85 866	3655	7374	0	832	1619	3891	106 470	3317	7152	0	550	1156	3441	105 220	<.01	.43
41-45 y	5115	8015	396	1365	2900	5737	73 949	4152	8467	74	864	1626	3859	95 963	3354	7999	0	582	1158	2939	86 899	<.01	.14
Inpatient care																							
Total (18-45 y)	1849	3543	0	180	414	2560	65 544	1348	3700	0	0	0	1778	92 705	1169	3606	0	0	0	900	96 021	<.01	.07
18-19 y	—	—	—	—	—	—	—	—	—	—	—	—	—	—	—	—	—	—	—	—	—	—	
20-26 y	1465	2497	0	0	352	1943	20 307	1065	3146	0	0	0	692	32 600	1044	3408	0	0	0	224	39 926	.06	.93
27-30 y	1759	3135	0	192	402	2621	39 695	1437	3419	0	0	0	2152	42 797	1321	2598	0	0	0	2347	21 958	<.01	.51
31-35 y	1724	3141	0	158	430	2515	56 132	1570	3593	0	0	0	2639	55 649	1281	3223	0	0	0	1850	40 996	<.01	.10
36-40 y	2031	4149	0	170	454	2641	50 691	1075	2857	0	0	0	775	43 195	1082	4697	0	0	0	108	96 021	<.01	.98
41-45 y	2241	4395	0	289	448	3135	65 544	1428	5226	0	0	0	772	92 705	998	3907	0	0	0	0	65 949	<.01	.16
Outpatient care																							
Total (18-45 y)	1418	1329	0	649	1046	1746	16 525	1339	1397	0	566	974	1663	24 805	1170	1912	0	410	775	1373	58 711	<.01	<.01
18-19 y	—	—	—	—	—	—	—	—	—	—	—	—	—	—	—	—	—	—	—	—	—	—	
20-26 y	1186	855	46	626	960	1469	6 017	1132	1020	73	544	855	1390	8 058	1079	1505	0	411	695	1143	13 922	.25	.58
27-30 y	1386	1219	89	651	1100	1700	13 701	1373	1268	0	610	1063	1692	13 563	1163	1423	0	436	859	1433	23 716	<.01	<.01
31-35 y	1526	1458	63	667	1167	1878	14 188	1409	1468	44	572	1032	1753	24 805	1254	2023	0	441	828	1572	45 887	<.01	.09
36-40 y	1451	1520	33	623	985	1728	16 525	1423	1635	0	555	973	1780	22 129	1092	1196	0	391	759	1280	11 195	<.01	<.01
41-45 y	1416	1261	0	652	983	1788	10 038	1231	1345	0	555	901	1510	20 770	1211	2984	0	381	700	1264	58 711	.17	.89
Pharmaceuticals^b^																							
Total (18-45 y)	674	4227	0	25	72	174	77 150	649	4124	0	26	74	189	76 377	639	4222	0	16	59	164	95 281	.76	.92
18-19 y	—	—	—	—	—	—	—	—	—	—	—	—	—	—	—	—	—	—	—	—	—	—	
20-26 y	592	4425	0	23	67	143	72 468	557	3653	0	21	54	123	40 776	238	1205	0	18	47	119	19 480	.15	.12
27-30 y	391	2346	0	23	64	138	42 608	602	3852	0	25	71	154	44 880	595	3534	0	14	55	148	52 638	.24	.97
31-35 y	813	4383	0	23	72	173	47 759	628	3811	0	26	72	185	61 081	1004	6515	0	18	62	172	95 281	.50	.17
36-40 y	826	5472	0	27	71	203	77 150	692	4792	0	27	83	222	76 377	491	2614	0	16	59	172	36 036	.19	.38
41-45 y	684	3927	0	30	90	224	48 451	765	4416	0	30	97	272	44 738	579	3138	0	16	73	205	46 471	.65	.46
Sick leave payments^c^																							
Total (18-45 y)	302	1913	0	0	0	302	47 398	169	1026	0	0	0	169	23 463	183	1482	0	0	0	0	25 235	<.01	.68
18-19 y	—	—	—	—	—	—	—	—	—	—	—	—	—	—	—	—	—	—	—	—	—	—	—
20-26 y	253	2031	0	0	0	302	34 881	52	216	0	0	0	0	3 821	68	514	0	0	0	0	7 474	.10	.58
27-30 y	239	1149	0	0	0	302	15 860	160	831	0	0	0	169	10 648	157	1658	0	0	0	0	25 235	.32	.97
31-35 y	205	1164	0	0	0	302	18 323	98	503	0	0	0	169	8 817	107	778	0	0	0	0	14 602	.05	.80
36-40 y	344	1941	0	0	0	302	24 687	177	1136	0	0	0	169	23 463	313	2050	0	0	0	0	23 688	.79	.17
41-45 y	533	3164	0	0	0	302	47 398	377	1805	0	0	0	169	19 425	268	1742	0	0	0	0	20 874	.12	.35
Aids and remedies^c^																							
Total (18-45 y)	202	726	0	0	90	202	26 299	249	1095	0	0	95	249	29 380	265	1223	0	0	89	265	40 286	.02	.60
18-19 y	—	—	—	—	—	—	—	—	—	—	—	—	—	—	—	—	—	—	—	—	—	—	—
20-26 y	154	755	0	0	69	202	13 845	266	1661	0	0	63	249	29 380	176	394	0	0	84	265	5 728	.64	.33
27-30 y	211	1143	0	0	81	202	26 299	179	532	0	0	64	249	8 904	197	634	0	0	50	265	10 314	.79	.60
31-35 y	200	559	0	0	88	202	9 959	206	649	0	0	93	249	10 675	284	1282	0	0	98	265	23 817	.10	.14
36-40 y	194	440	0	0	95	202	6 493	287	1209	0	0	111	249	25 193	339	1951	0	0	60	265	40 286	.08	.59
41-45 y	242	510	0	0	136	202	5 292	351	1478	0	0	152	249	26 476	299	886	0	0	119	265	12 302	.23	.52

Abbreviations: Max, maximum; Med, median; Min, minimum; Q1, 25th percentile; Q3, 75th percentile.

Study cohort 1: women with CIN diagnoses and a subsequent cervical conization; study cohort 2: women with CIN diagnoses and no cervical conization in the entire study period; control group: women with neither a CIN diagnosis nor a conization in the entire study period.

^a^Compared with control group with t test. Due to data protection regulations, no analysis of age group 18-19 was possible, as patient counts were 1-4.

^b^Reflecting costs for outpatient pharmaceuticals; costs for inpatient pharmaceuticals are included in inpatient costs.

^c^Imputed: As information on sick leave costs and aids and remedies costs are not complete for all individuals in the InGef Research Database, missing sick leave and aids and remedies data were imputed by using the mean sick leave costs of women with available sick leave data and aids and remedies data, respectively.

The increment of inpatient costs was €680 (*P<*.01) and €179 (*P=*.07) in study cohort 1 and 2 compared with the control group, respectively. The highest inpatient cost difference in study cohort 1 (€1243, *P<*.01) and study cohort 2 (€430, *P=*.16) compared with the control groups was observed in the 41-45 age group (Table 2). In the outpatient setting, the highest cost difference in study cohort 1 (€359, *P<*.01) and study cohort 2 (€331, *P<*.01) compared with the control group was observed in the 31-35 age group.

After winsorization, mean healthcare costs in all domains were lower in all cohorts. Tendencies regarding differences between study cohort 1 and the control group remained as before winsorization. In study cohort 2, however, more significant differences were observed after winsorization. Winsorized cost results can be found in the **Supplement, Section 4, Table 3**.

### HRU During 24-Month Follow-up

During 24 months’ follow-up, significantly more women were hospitalized at least once in study cohort 1 (56.46%, *P<*.01) and study cohort 2 (38.74%, *P<*.01) compared with the control group (31.14%) (Table 3). The age groups with the highest differences with respect to the proportion of women with at least 1 hospitalization compared with the control group were the 36-40 age group in study cohort 1 and the 31-35 age group in study cohort 2.

**Table 3.** Proportions of Women 18-45 With HRU During 24-Month Follow-up, Stratified by Age

	**n (%)**			***P* Value^a^**	
	**Study Cohort 1**	**Study Cohort 2**	**Control Group**	**Study Cohort 1**	**Study Cohort 2**
Hospitalizations					
Total (18-45 y)	1552 (56.46)	1065 (38.74)	856 (31.14)	<.01	<.01
18-19 y	<5	0 (0)	0 (0)	—	—
20-26 y	179 (51.88)	133 (38.55)	98 (28.41)	<.01	<.01
27-30 y	340 (56.86)	245 (40.97)	230 (38.46)	<.01	<.01
31-35 y	434 (56.81)	348 (45.55)	264 (34.55)	<.01	<.01
36-40 y	326 (56.50)	186 (32.24)	151 (26.17)	<.01	<.01
41-45 y	270 (58.57)	153 (33.19)	113 (24.51)	<.01	<.01
Outpatient visits					
Total (18-45 y)	2748 (99.96)	2748 (99.96)	2713 (98.69)	<.01	<.01
18-19 y	<5	<5	<5	—	—
20-26 y	345 (100.00)	345 (100.00)	341 (98.84)	.12	.04
27-30 y	598 (100.00)	598 (100.00)	588 (98.33)	<.01	<.01
31-35 y	764 (100.00)	764 (100.00)	758 (99.21)	.03	<.01
36-40 y	577 (100.00)	576 (99.83)	568 (98.44)	<.01	<.01
41-45 y	460 (99.78)	461 (100.00)	454 (98.48)	.07	<.01

Study cohort 1: women with CIN diagnoses and a subsequent cervical conization; study cohort 2: women with CIN diagnoses and no cervical conization in the entire study period; control group: women with neither a CIN diagnosis nor a conization in the entire study period.

^a^Compared with control group with χ^2^ test or Fisher’s test where applicable. Due to data protection regulations, no analysis of age group 18-19 was possible, as patient counts were 1-4.

Women in study cohort 1 (mean, 1.25 hospitalizations; *P<*.01) and study cohort 2 (mean, 0.92 hospitalizations; *P<*.01), were significantly more frequently hospitalized than women in the control group (mean, 0.72 hospitalizations) (Table 4). Women in study cohort 1 had on average almost twice as many hospitalizations as women in the control group. The highest mean difference concerning frequency of hospitalizations was observed for the 41-45 age group (1.25 vs 0.50 hospitalizations, *P<*.01) in study cohort 1 and the 31-35 age group (1.12 vs 0.82 hospitalizations, *P<*.01) in study cohort 2.

**Table 4.** Frequency of HRU in Women 18-45 During 24-Month Follow-up, Stratified by Age

	**Study Cohort 1**							**Study Cohort 2**							**Control Group**							***P* Value^a^**	
	**Mean**	**SD**	**Min**	**Q1**	**Med**	**Q3**	**Max**	**Mean**	**SD**	**Min**	**Q1**	**Med**	**Q3**	**Max**	**Mean**	**SD**	**Min**	**Q1**	**Med**	**Q3**	**Max**	**Study Cohort 1**	**Study Cohort 2**
Hospitalizations																							
Total (18-45 y)	1.25	1.78	0	0	1	2	15	0.92	1.75	0	0	0	1	21	0.72	1.52	0	0	0	1	17	<.01	<.01
18-19 y	—	—	—	—	—	—	—	—	—	—	—	—	—	—	—	—	—	—	—	—	—	—	
20-26 y	1.13	1.64	0	0	1	2	12	0.90	1.64	0	0	0	1	10	0.69	1.64	0	0	0	1	17	<.01	.09
27-30 y	1.33	1.86	0	0	1	2	13	0.98	1.76	0	0	0	2	21	0.90	1.59	0	0	0	2	14	<.01	.44
31-35 y	1.32	1.81	0	0	1	2	14	1.12	1.80	0	0	0	2	18	0.82	1.49	0	0	0	1	11	<.01	<.01
36-40 y	1.15	1.66	0	0	1	2	12	0.79	1.89	0	0	0	1	20	0.61	1.58	0	0	0	1	17	<.01	.08
41-45 y	1.25	1.86	0	0	1	2	15	0.71	1.52	0	0	0	1	11	0.50	1.25	0	0	0	0	13	<.01	.02
Length of stay (days)																							
Total (18-45 y)	7.19	33.04	0	0	1	5	594	7.27	37.34	0	0	0	4	581	5.86	39.30	0	0	0	2	1008	.18	.17
18-19 y	—	—	—	—	—	—	—	—	—	—	—	—	—	—	—	—	—	—	—	—	—	—	
20-26 y	5.74	22.46	0	0	1	4	328	7.82	37.01	0	0	0	2	523	6.21	40.23	0	0	0	1	598	.85	.58
27-30 y	8.22	39.63	0	0	1	5	535	7.55	35.19	0	0	0	4	488	6.61	33.05	0	0	0	5	484	.45	.63
31-35 y	6.70	30.15	0	0	1	5	547	7.09	33.46	0	0	0	5	406	4.57	21.86	0	0	0	3	473	.11	.08
36-40 y	7.30	35.71	0	0	1	5	594	5.52	30.49	0	0	0	2	482	8.05	64.30	0	0	0	1	1008	.81	.39
41-45 y	7.64	31.59	0	0	1	5	395	9.05	51.64	0	0	0	2	581	4.08	24.79	0	0	0	0	415	.06	.06
Outpatient visits																							
Total (18-45 y)	43.23	26.19	0	25	37	53	234	38.60	26.16	0	21	33	50	224	32.07	33.88	0	14	25	41	696	<.01	<.01
18-19 y	—	—	—	—	—	—	—	—	—	—	—	—	—	—	—	—	—	—	—	—	—	—	
20-26 y	40.59	21.13	4	25	38	51	138	35.26	22.34	1	20	32	45	143	29.79	25.58	0	14	24	36	235	<.01	<.01
27-30 y	42.31	23.28	4	25	38	53	153	39.07	26.47	1	22	34	49	224	32.88	36.37	0	16	26	42	696	<.01	<.01
31-35 y	44.48	26.72	6	26	38	56	187	39.96	27.64	1	21	33	50	218	33.30	33.86	0	14	26	43	655	<.01	<.01
36-40 y	43.54	29.57	7	24	36	53	234	38.69	25.94	0	21	32	51	179	30.56	26.18	0	13	24	42	272	<.01	<.01
41-45 y	43.95	27.70	0	27	37	53	187	38.19	26.15	1	20	33	50	176	32.63	43.34	0	13	23	39	547	<.01	.02

Abbreviations: Min, minimum; Q1, 25th percentile; Med, Median; Q3, 75th percentile; Max, maximum.

Study cohort 1: women with CIN diagnoses and a subsequent cervical conization; study cohort 2: women with CIN diagnoses and no cervical conization in the entire study period; control group: women with neither a CIN diagnosis nor a conization in the entire study period.

^a^Compared with control group using *t* test. Due to data protection regulations, no analysis of age group 18-19 was possible, as patient counts were n=1-4.

Even though the mean overall length of hospital stays (Table 4) for women aged 18-45 years was higher in study cohort 1 (7.19 days, *P=*.18) and study cohort 2 (7.27 days, *P=*.17) compared with the control group (5.86 days); this difference was not statistically significant. No significant differences of length of hospital stay were found in any age group in the 2 study cohorts compared with the control group.

The mean frequency of outpatient physician visits in women aged 18-45 years was significantly higher in study cohort 1 (43.23 visits, *P<*.01) and study cohort 2 (38.60 visits, *P<*.01) compared with the control group (32.07 visits) (Table 4). The highest mean difference of 12.98 visits (*P<*.01) between study cohort 1 and the control group was observed for women aged 36-40 years. In study cohort 2, the highest mean difference was observed for the age group 36-40 years compared with controls (8.13 visits, *P<*.01).

## DISCUSSION

We assessed the healthcare costs and HRU of women with CIN who underwent cervical conization (study cohort 1) and women with CIN of the same severity grade as study cohort 1 but who did not undergo conization (study cohort 2) during a 24-month follow-up.

We found 30% and 10% higher total healthcare costs in study cohort 1 and study cohort 2, respectively, compared with the control group. Highest cost differences between study cohort 1 and controls were found in the 41-45 (€1761, *P<*.01) and 36-40 (€1529, *P<*.01) age groups. In study cohort 2, the highest mean differences were observed for age groups 41- 45 (€798, *P=*.14) and 20-26 years (€468, *P=*.31) but were not statistically significant. Cost differences between both study cohorts and the control group were mainly driven by inpatient and outpatient care, with inpatient care being the main cost driver for study cohort 1 and equally high mean costs in inpatient and outpatient care in study cohort 2. Women in both study cohorts presented with more hospitalizations and outpatient physician contacts than the control group during the 2-year follow-up period.

This study is the first to report age-stratified healthcare costs and HRU associated to CIN. In addition to the expected higher costs for women with CIN (in both study cohorts) compared with a control group of women without CIN, we also found differences between age groups. The highest differences in mean total healthcare costs in both study cohorts, compared with the control group, were observed in the age group 41-45 years (study cohort 1, €1761; *P<*.01; study cohort 2, €798, *P=*.14). The second highest differences in mean total healthcare costs were observed in different age groups in study cohort 1 (36-40 years: €1529; *P<*.01) and study cohort 2 (20-26 years: €468, *P=*.31). We did not test for differences between the age groups; however, our results indicate that CIN is associated with increased healthcare expenditures in the 36-45 and 20-26 age groups.

To date, data on costs of invasive treatment and conservative management of CIN from the perspective of the healthcare system are scarce. The few available studies are not readily comparable to our study due to different methodological approaches and differences in price levels across years and purchasing power between countries. In France, the total treatment cost for CIN (from a healthcare provider perspective) was estimated at €22.3 million in 2004.^27^ In Spain, the estimated annual total costs of CIN management were even higher, at €147 million; the mean estimated cost of CIN management per patient was €1115 for CIN1, €1626 for CIN2, and €2090 for CIN3 in 2009.^28^ However, this study did not distinguish between treated (conization) and untreated women for cost estimates. A more recent study from Spain estimated annual direct costs of CIN2/3 attributable to HPV types targeted by 9-valent vaccine (6/11/16/18/31/33/45/52/58) of €64.22 million for 27 648 cases with CIN2/3 in Spain in 2017, which corresponds to about €2322 annual costs or €4644 biannual direct costs per patient.^29^ In our study we found cost differences of €1020, which can be associated with CIN treatment for those women with CIN who underwent invasive treatment during a 2-year follow-up period. The considerable differences between the cost estimates from Spain and our results may be explained by differences in the study designs. Whereas our study focused on a female population aged 18-45 years, the Spanish study included results from other studies, presumably including all age groups, with more cost data for women older than 45 years. Additionally, only cost results for CIN2 and 3 were considered in the Spanish study, whereas our study included also around 4% of women with CIN1. The costs reported for Spain were based on a literature review including published literature from 1995-2017, which reported direct costs for the Spanish National Health Service. Due to these differences and potential differences between the German and Spanish health insurance systems, it is unclear how comparable the cost data from Spain are to our data. Data from German medical records of 138 patients with diagnosed abnormal Pap test results from 2004-2005 estimated an average annual cost for managing and treating precancerous cervical lesions of about €943-€3174.^22^ Although this cost estimate seems closer to our estimated cost of €1020 associated with invasive treatment in women with CIN, our results are still lower. The same German study, using medical health records, reported a hospitalization rate of 28.3% for the assessed sample.^22^ In our study, we found hospitalization rate differences of 25.3% in women with invasive treatment and 7.6% in women with no documented intervention, compared with the control group. The differences in healthcare costs and HRU might be explained by the different study designs and study populations, as the referenced study was based only on a small, presumably older, specifically selected sample of women with abnormal Pap test results, which were detected during cervical cancer screening and closely followed up by their gynecologists. This might have had an impact on the documentation in the medical records, which were evaluated for cost and resource estimates. Our study, on the other hand, used claims data from the SHI in Germany, which makes a cost comparison between the two studies difficult.

Furthermore, it must be kept in mind that our analysis captures only a snapshot of costs and HRU associated with CIN management. Costs were not assessed beginning from the date of an incident CIN diagnosis but from the date of initial conization in study cohort 1, from which a virtual index date for women without conization was calculated. This is because incidence is impossible to identify for a condition that is usually asymptomatic and identified only by opportunistic screening and in cases where only a restricted period of claims data is available. This is the case for Germany, where it is not allowed by law to analyze more than 6 consecutive years of claims data. The costs of possibly long-lasting and in-depth clinical and cytologic/histologic evaluation to confirm the initial diagnosis before the index conization procedure or the start of the follow-up period in women without conization were therefore not included in the analysis, suggesting that per-case cost may be considerably higher. Additionally, the 24-month follow-up period might have also limited the observed costs and HRU given a possible natural history of CIN progression over several years.

As 45.5% and 82.3% of CIN2+ cases are estimated to be attributable to the HPV types targeted by the quadrivalent vaccine (HPV6/11/16/18) and 9-valent vaccine, respectively,^30^ we hypothesize that parts of the estimated costs and HRU associated to CIN management in this study would be preventable by HPV vaccination.

To interpret the results for study cohort 2, it must be considered that these women were matched to study cohort 1 based on most severe CIN grade in the baseline period. Thus, cohort 2 does not represent all women with CIN who did not undergo conization but specifically untreated women with CIN grades of the same severity as women that underwent conization. We found on average higher baseline CIN grades in women eligible for study cohort 1 as compared with women eligible for study cohort 2 (indicating that women with higher CIN grades are more likely to undergo conization). The CIN grade distribution in study cohort 1 was retained after matching. In contrast, the CIN grade distribution in study cohort 2 after matching reflected the CIN grade distribution in study cohort 1. We assume that higher CIN grades in women in study cohort 2 are overrepresented in our analyses and do not necessarily reflect the actual distribution of CIN grades among women with conservative management, underestimating the frequency of women with CIN1 and conservative management and thereby making the study cohort 2 “sicker” than their population of origin. On the other hand, women in study cohort 2 were not allowed to undergo any conization in the complete study period of 6 years, and women in the control group were not allowed to have any CIN diagnosis or conization records within the complete study period. This approach may have produced 2 cohorts that were artificially made “healthier.” Specifically, study cohort 2 can be assumed to reflect the HRU and costs of women with CIN that was successfully managed with a conservative approach. Although we consider both our study cohorts and the control group as well balanced in terms of described characteristics, the application of any matching approach cannot fully eliminate the risk of residual confounding. Potential group imbalances may also remain with regard to unobserved confounders. Thus, some uncertainty with regard to causal conclusions remains and must be considered when interpreting the results.

Women whose CIN was initially managed conservatively with a decision for conization at a later time were not considered. Consequently, healthcare costs and HRU of women with a CIN diagnosis who start on conservative management and progressed to invasive treatment as well as initially healthy women may not be reported in this study. As we wanted to assess the cost and resource use of women with CIN grades that would have warranted conization but who were managed conservatively without conization, this was a fair approach.

Considering the limitations of our study populations, the validity of our control group is central for the validity of the observed increments. For the control group, we were able to externally validate the representativeness of their HRU and did not find indications that this group was artificially made healthier through our selection algorithm. In 2017, another source assessing German SHI claims data reports an annual mean frequency of days with documented outpatient services in women aged 20-44 years of 13.0-15.3 days, resulting in an estimated 26.0-30.6 days with physician visits during 2 years of follow-up.^31^ In our control group, we found slightly higher mean physician contacts (32.1 visits). Both of our study cohorts showed higher mean frequencies for outpatient physician visits than the control group. This was expected due to the regular follow-up also needed for women with conservative management visits according to the guideline.^18^ With a validated control group, it is reasonable to assume that the differences observed between both study groups—even with their limitations—are associated to the management of CIN, covering all healthcare domains reimbursed by the SHI in Germany. This is a major strength of the selected study design as it highlights the cost increase, which can be directly associated to CIN.

A further limitation related to the use of claims data is the availability of codes used for the identification of the study population, which ultimately depends on the codes documented by the physicians. For the identification of women with CIN and surgical treatment, we focused on specific OPS codes for cervical conization (5-671.0* or 5-671.1*), as this is the recommended procedure for removal of CIN in Germany.^18^ However, the German OPS catalog contains an additional code for “other excision of cervix” (OPS code 5-672*). In cases where this code was documented instead of the recommended procedure, women would not have been identified as eligible for study group 1, and consequently healthcare costs and HRU in these women might not be captured. Also, women with this code were not excluded from study cohort 2 and the control group. For women in the control group, this might be a negligible risk, as these women were not allowed to have any documentation for CIN or cervical cancer over the entire 6-year study period. For study cohort 2, however, it is possible that this code was documented instead of the code for cervical conization. Hence, it is possible that a few of these women might have been surgically treated for their CIN, even though the specific code for cervical conization was not documented.

## CONCLUSION

Our results showed higher HRU in women aged 18-45 years with CIN diagnoses undergoing invasive treatment (study cohort 1) or conservative management (study cohort 2) compared with women without CIN (control group) in a 2-year follow- up period, leading to higher total healthcare costs of €1020 and €328, respectively. The highest incremental healthcare costs were found in women between 36 and 45 years and were mainly driven by costs for inpatient care. Further research to understand the full follow-up costs of CIN diagnoses is necessary. Intensified efforts to prevent CIN, conization, and associated costs will be important, as may extended HPV vaccination in adolescents and adult catch-up cohorts.

### Declarations

MR and RW are full-time employees of MSD Sharp & Dohme GmbH. AJS was a full-time employee of MSD Sharp & Dohme GmbH at the time the study was conducted. KS and VSP are full-time employees of Merck Sharp & Dohme LLC, a subsidiary of Merck & Co, Inc, Rahway, New Jersey. CJ and KMS are full-time employees of Xcenda GmbH, acting as contractors of Merck Sharp & Dohme Corp, a subsidiary of Merck & Co, Inc, Rahway, New Jersey, for the execution of this study. WG received personal fees from Xcenda GmbH during the conduct of the study. MH received honoraria as speaker and member of medical advisory boards from MSD Sharp & Dohme GmbH.

### Ethics Approval and Consent

This study used anonymized German claims data. Therefore, no ethics approval was needed by an independent ethics committee or institutional review board. The utilized database addresses all data protection regulations in Germany. To ensure the protection of individual data and privacy, regions that are smaller than federal states or patient cohorts with less than 100 individuals were not analyzed in a granular way. Furthermore, patient counts below 5 were reported as “<5.” For this type of study, formal consent is not required.

### Availability of Data and Material

The data used in this study cannot be made available in the manuscript, the supplemental files, or in a public repository due to German data protection laws (Bundesdatenschutzgesetz). To facilitate the replication of results, anonymized data used for this study are stored on a secure drive at the Institute for Applied Health Research Berlin (InGef). Access to the data used in this study can be provided to external parties only under the conditions of the cooperation contract of this research project and can be assessed upon request, after written approval (info@ingef.de).

### Code Availability

Not available.

## Supplementary Material

Online Supplementary Material

## References

[ref-120828] Ferris Daron G., Brown Darron R., Giuliano Anna R., Myers Evan, Joura Elmar A., Garland Suzanne M., Kjaer Susanne K., Perez Gonzalo, Saah Alfred, Luxembourg Alain, Velicer Christine (2020). Prevalence, incidence, and natural history of HPV infection in adult women ages 24 to 45 participating in a vaccine trial. Papillomavirus Research.

[ref-120829] Petry Karl Ulrich, Luyten Alexander, Justus Annika, Iftner Angelika, Strehlke Sarah, Reinecke-Lüthge Axel, Grunwald Elisabeth, Schulze-Rath Renate, Iftner Thomas (2013). Prevalence of high-risk HPV types and associated genital diseases in women born in 1988/89 or 1983/84 – results of WOLVES, a population-based epidemiological study in Wolfsburg, Germany. BMC Infectious Diseases.

[ref-120830] Plummer Martyn, Schiffman Mark, Castle Philip E., Maucort-Boulch Delphine, Wheeler Cosette M. (2007). A 2-year prospective study of human papillomavirus persistence among women with a cytological diagnosis of atypical squamous cells of undetermined significance or low-grade squamous intraepithelial lesion. The Journal of Infectious Diseases.

[ref-120831] Centers for Disease Control and Prevention (2020). Human papillomavirus (HPV).

[ref-120832] Skinner S. Rachel, Wheeler Cosette M., Romanowski Barbara, Castellsagué Xavier, Lazcano-Ponce Eduardo, Rowena Del Rosario-Raymundo M., Vallejos Carlos, Minkina Galina, Pereira Da Silva Daniel, McNeil Shelly, Prilepskaya Vera, Gogotadze Irina, Money Deborah, Garland Suzanne M., Romanenko Viktor, Harper Diane M., Levin Myron J., Chatterjee Archana, Geeraerts Brecht, Struyf Frank, Dubin Gary, Bozonnat Marie-Cécile, Rosillon Dominique, Baril Laurence, for the VIVIANE study group (2016). Progression of HPV infection to detectable cervical lesions or clearance in adult women: analysis of the control arm of the VIVIANE study. International Journal of Cancer.

[ref-120833] Demarco Maria, Hyun Noorie, Carter-Pokras Olivia, Raine-Bennett Tina R., Cheung Li, Chen Xiaojian, Hammer Anne, Campos Nicole, Kinney Walter, Gage Julia C., Befano Brian, Perkins Rebecca B., He Xin, Dallal Cher, Chen Jie, Poitras Nancy, Mayrand Marie-Helene, Coutlee Francois, Burk Robert D., Lorey Thomas, Castle Philip E., Wentzensen Nicolas, Schiffman Mark (2020). A study of type-specific HPV natural history and implications for contemporary cervical cancer screening programs. EClinicalMedicine.

[ref-120834] McCredie Margaret RE, Sharples Katrina J, Paul Charlotte, Baranyai Judith, Medley Gabriele, Jones Ronald W, Skegg David CG (2008). Natural history of cervical neoplasia and risk of invasive cancer in women with cervical intraepithelial neoplasia 3: a retrospective cohort study. The Lancet Oncology.

[ref-120835] Robert Koch-Institute (2007). German Standing Committee on Vaccination: Mitteilung der Ständigen Impfkommission (STIKO) am Robert Koch-Institut: Impfung gegen humane Papillomaviren (HPV) für Mädchen von 12 bis 17 Jahren: Empfehlung und Begründung. Epidemiol Bull.

[ref-120836] Robert Koch-Institute (2014). German Standing Committee on Vaccination: Mitteilung der Ständigen Impfkommission (STIKO) am Robert Koch-Institut: Empfehlungen der Ständigen Impfkommission (STIKO) am Robert Koch- Institut. Epidemiol Bull.

[ref-120837] Robert Koch-Institute (2018). German Standing Committee on Vaccination: Wissenschaftliche Begründung für die Empfehlung der HPV-Impfung für Jungen im Alter von 9 bis 14 Jahren. Epidemiol Bull.

[ref-120838] Klug Stefanie J., Hukelmann Meike, Hollwitz Bettina, Düzenli Nurgül, Schopp Betti, Petry Karl-Ulrich, Iftner Thomas (2007). Prevalence of human papillomavirus types in women screened by cytology in Germany. Journal of Medical Virology.

[ref-120839] Bundesministerium für Gesundheit (2020). Weiterentwicklung der Gebärmutterhals-Krebsfrüherkennung.

[ref-120840] Gemeinsamer Bundesausschuss (2018). Versicherteninformation Gebärmutterhalskrebs-Früherkennung.

[ref-120841] Marquardt K., Kossowski I., Pfandzelter R. (2015). Jahresstatistik Zervix-Zytologie. Frauenarzt.

[ref-120842] Marquardt Katrin, Kossowski Izabella, Hantschke-Zerbich Heike, Michel Frank (2019). An der Schwelle zum organisierten Zervixkarzinomscreening. Der Gynäkologe.

[ref-120843] Reuschenbach Miriam, Mihm Sarah, Wölle Regine, Schneider Kim Maren, Jacob Christian, Braun Sebastian, Greiner Wolfgang, Hampl Monika (2020). Burden of HPV related anogenital diseases in young women in Germany—an analysis of German statutory health insurance claims data from 2012 to 2017. BMC Infectious Diseases.

[ref-120844] Kaatsch P., Spix C., Katalinic A.. (2015). Krebs in Deutschland 2011/2012; vol 10.

[ref-120845] Deutsche Krebsgesellschaft, Deutsche Krebshilfe, AWMF (2020). Leitlinienprogramm Onkologie: Präventiondes Zervixkarzinom.

[ref-120846] Cooper D.B., Menefee G.W. (2019). StatPearls.

[ref-120847] Poethko-Müller C., Buttmann-Schweiger N., Takla A. (2018). Impfung gegen Humane Papillomviren (HPV) von Mädchen in Deutschland – Querschnittergebnisse aus KiGGS Welle 2 und Trends. J Health Monit.

[ref-120848] Mühlhause I., Filz M. (2008). Screening auf Zervixkarzinom arznei-telegramm.

[ref-120849] Petry K.U., Breugelmans J.G., Bénard S., Lamure E., Littlewood K.J., Hillemanns P. (2008). Cost of screening and treatment of cervical dyskaryosis in Germany. Eur J Gynaecol Oncol.

[ref-120850] Statistisches Bundesamt DESTATIS (2019). Ergebnisse der Bevölkerungsfortschreibung auf Grundlage des Zensus 2011.

[ref-120851] Bundesministerium für Gesundheit (2020). Gesetzliche Krankenversicherung - Kennzahlen und Faustformeln.

[ref-120852] Andersohn Frank, Walker Jochen (2016). Characteristics and external validity of the German Health Risk Institute (HRI) Database. Pharmacoepidemiology and Drug Safety.

[ref-120853] Quan H., Li B., Couris C. M., Fushimi K., Graham P., Hider P., Januel J.-M., Sundararajan V. (2011). Updating and validating the Charlson comorbidity index and score for risk adjustment in hospital discharge abstracts using data from 6 countries. American Journal of Epidemiology.

[ref-120854] Bergeron C., Breugelmans J.-G., Bouée S., Lorans C., Bénard S., Rémy V. (2006). [Cervical cancer screening and associated treatment costs in France]. Gynécologie Obstétrique & Fertilité.

[ref-120855] Castellsagué Xavier, Rémy Vanessa, Puig-Tintoré Luis M., de la Cuesta Ricardo Sainz, Gonzalez-Rojas Nuria, Cohet Catherine (2009). Epidemiology and costs of screening and management of precancerous lesions of the cervix in Spain. Journal of Lower Genital Tract Disease.

[ref-120856] López Noelia, Torné Aureli, Franco Agustín, San-Martin María, Viayna Elisabet, Barrull Carmen, Perulero Nuria (2018). Epidemiologic and economic burden of HPV diseases in Spain: implication of additional 5 types from the 9-valent vaccine. Infectious Agents and Cancer.

[ref-120857] Hartwig Susanne, Baldauf Jean-Jacques, Dominiak-Felden Géraldine, Simondon François, Alemany Laia, de Sanjosé Silvia, Castellsagué Xavier (2015). Estimation of the epidemiological burden of HPV-related anogenital cancers, precancerous lesions, and genital warts in women and men in Europe: potential additional benefit of a nine-valent second generation HPV vaccine compared to first generation HPV vaccines. Papillomavirus Research.

[ref-120858] Grobe T., Steinmann S., Szecsenyi J. (2019). BARMER Arztreport 2019—Schriftenreihe zur Gesundheitsanalyse. Vol Band 14.

