# Supplementary material for: Healthcare Costs and Resource Use Associated With Cervical Intraepithelial Neoplasia and Cervical Conization: A Retrospective Study of German Statutory Health Insurance Claims Data
Source: J Health Econ Outcomes Res. 2022 May 26;9(1):128–39. doi: 10.36469/001c.35329 (PMC9135472; doi:10.36469/001c.35329)
Supplement: Online Supplementary Material [file jheor_2022_9_1_35329_91157.pdf]

JHEOR

Journal of Health Economics and Outcomes Research

Online Supplementary Material

Healthcare Costs and Resource Use Associated With Cervical Intraepithelial Neoplasia and Cervical Conization: A Retrospective Study of German Statutory Health Insurance Claims Data. *JHEOR*. 2022;9(1):128-139. doi:10.36469/jheor.2022.35329

1. **Data Domains Included in the Institute for Applied Health Research Berlin (InGef) Database**
2. **Definitions of Baseline Demographics and Clinical Characteristics (Table S1: ICD-10-GM Codes for the Assessment of Clinical Characteristics in the Baseline Period)**
3. **Identification of Cervical Conization (Table S2: OPS Codes Utilized for Identification of Cervical Conization)**
4. **All-Cause Healthcare Costs After Winsorization (Table S3: All-Cause Healthcare Costs During 24-Month Follow-up, Stratified by Age, After Winsorization)**

This supplementary material has been provided by the authors to give readers additional information about their work.

This is an open-access article distributed under the terms of the Creative Commons Attribution 4.0 International License (CCBY-4.0). View this license's legal deed at http://creativecommons.org/licenses/by/4.0 and legal code at http://creativecommons.org/licenses/by/4.0/legalcode for more information.

1. DATA DOMAINS INCLUDED IN THE INSTITUTE FOR APPLIED HEALTH RESEARCH BERLIN (INGEF) DATABASE

The included data domains in the InGef database cover the following:

- Patient demographics such as age, gender, insurance status (eg, retired, family insurance), and the region of residence
- Outpatient care including dates of outpatient visits and respective specialty, performed procedures (eg, laboratory services, radiology, echocardiography) as well as the documented diagnoses as *International Statistical Classification of Diseases, 10th Revision, German Modification* (ICD--10-GM) codes
- Inpatient care summarizing hospital admission information such as admission and discharge dates, ICD-10-GM diagnoses, and German Diagnosis Related Groups (G-DRG) codes
- Prescribed outpatient pharmaceuticals on package level
- Prescribed aids and remedies with type of remedy, device, or aid with the respective code and description
- Incapacity to work and sick leave payments, including ICD-10-GM diagnoses leading to the sick leave episode, duration of sick leave, and start/end dates of sick leave payments

2. DEFINITION OF BASELINE DEMOGRAPHICS AND CLINICAL CHARACTERISTICS

Baseline demographic and baseline clinical characteristics were identified during the 6-month period before the (virtual) index event and consist of the following:

Baseline Demographics

Age was determined in the quarter of highest cervical intraepithelial neoplasia (CIN) grade diagnosis in the 6-month baseline period (both study cohorts); for the control group, age was determined in the quarter used for the matching, thus, age of control group could only be determined after the matching was performed. The results were further stratified by the following age groups: 18-19 years, 20-26 years, 27-30 years, 31-35 years, 36-40 years, and 41-45 years.

- Year of conization (only for study cohort 1)
- Conization type at index (only for study cohort 1)
- Geographic region of living was determined as the last documented living place before the index date:
    - North (Schleswig-Holstein, Hamburg, Bremen, Lower Saxony, Mecklenburg-Western Pomerania)
    - East (Thuringia, Brandenburg, Berlin, Saxony, Saxony-Anhalt)
    - West (North Rhine, Saarland, Rhineland-Palatinate, Hesse)
    - South (Bavaria, Baden-Württemberg)
- Living place (urban or rural living place) was determined as the last documented living place before the index date
- Insurance member type was determined as the last documented member type before the index date:
    - Member
    - Dependent coverage
    - Retired

Clinical Characteristics

Associated diagnoses were identified as ICD-10-GM codes in the inpatient sector (main or secondary diagnosis) or outpatient sector (verified diagnosis) recorded in the respective baseline period. The reported CIN diagnoses are the highest severity grade recorded during the baseline period, ultimately being the CIN diagnosis used as a matching parameter for study cohort 1 and study cohort 2.

The Charlson Comorbidity Index (CCI)[1] score was calculated for both study cohorts as well as the matched controls and reported as number and proportions of patients in the CCI groups (0, 1, 2, 3, and 4+).

[1]Quan H, Li B, Couris CM, et al. Updating and validating the Charlson comorbidity index and score for risk adjustment in hospital discharge abstracts using data from 6 countries. *Am J Epidemiol.* 2011;173(6):676-682.

Table S1. ICD-10-GM Codes for the Assessment of Clinical Characteristics in the Baseline Period

ICD-10-GM Code	Description	German Description
N87.0	CIN I	Niedriggradige Dysplasie der Cervix uteri<br>Niedriggradige squamöse intraepitheliale Läsion [LSIL]<br>Zervikale intraepitheliale Neoplasie [CIN] I. Grades
N87.1	CIN II	Mittelgradige Dysplasie der Cervix uteri<br>Zervikale intraepitheliale Neoplasie [CIN] II. Grades
D06.-	CIN III	Carcinoma in situ der Cervix uteri<br>Zervikale intraepitheliale Neoplasie [CIN] III. Grades, mit oder ohne Angabe einer hochgradigen Dysplasie
N87.2		Hochgradige Dysplasie der Cervix uteri, anderenorts nicht klassifiziert
C53.-	Cervical cancer	Bösartige Neubildung der Cervix uteri

Abbreviations: CIN, cervical intraepithelial neoplasia, ICD-10-GM, *International Classification of Diseases, 10th Revision, German Modification*; LSIL, low-grade squamous intraepithelial lesion [niedriggradige squamöse intraepitheliale Läsion].

3. IDENTIFICATION OF CERVICAL CONIZATION

Table 2. OPS Codes Utilized for Identification of Cervical Conization

OPS Code	Description
5-671.0	Conization
5-671.00	Laser excision
5-671.01	Loop excision
5-671.02	Knife excision
5-671.03	Electrical needle/knife excision
5-671.0x	Other

Abbreviation: OPS, German classification of operation and procedures [Operations- und Prozedurenschlüssel].

4. ALL-CAUSE HEALTHCARE COSTS AFTER WINSORIZATION

Table 3. All-Cause Healthcare Costs During 24-Month Follow-up, Stratified by Age, After Winsorization

	Costs (€)																					*P* Value[a]	
	Study Cohort 1							Study Cohort 2							Control Group								
	Mean	SD	Min	Q1	Med	Q3	Max	Mean	SD	Min	Q1	Med	Q3	Max	Mean	SD	Min	Q1	Med	Q3	Max	Study Cohort 1	Study Cohort 2
Total costs																							
Total (18-45 y)	4041	4597	74	1297	2368	5107	24843	3358	4299	0	851	1677	4292	23316	3048	4702	0	591	1238	3755	26132	<.01	.01
18-19 y	—	—	—	—	—	—	—	—	—	—	—	—	—	—	—	—	—	—	—	—	—	—	—
20-26 y	3403	4146	280	1233	1967	3998	24843	2678	3740	82	745	1320	3011	23316	2400	3612	0	588	1065	2819	26132	<.01	.32
27-30 y	3781	4093	128	1204	2207	5160	24843	3444	4223	39	852	1792	4489	23316	3161	4473	0	639	1429	4333	26132	.01	.26
31-35 y	4155	4564	74	1411	2453	5264	24843	3587	4175	52	894	1908	4901	23316	3366	4982	0	610	1336	4480	26132	<.01	.35
36-40 y	4140	4886	113	1249	2338	4927	24843	3246	4175	0	832	1619	3891	23316	3013	4858	0	550	1156	3441	26132	<.01	.38
41-45 y	4556	5145	396	1365	2900	5737	24843	3535	5049	74	864	1626	3859	23316	2920	5010	0	582	1158	2939	26132	<.01	.06
Inpatient care																							
Total (18-45 y)	1674	2302	0	180	414	2560	9953	1153	2117	0	0	0	1778	9395	990	2012	0	0	0	900	9410	<.01	<.01
18-19 y	—	—	—	—	—	—	—	—	—	—	—	—	—	—	—	—	—	—	—	—	—	—	—
20-26 y	1386	2073	0	0	352	1943	9953	879	1854	0	0	0	692	9395	845	1894	0	0	0	224	9410	<.01	.81
27-30 y	1611	2176	0	192	402	2621	9953	1262	2211	0	0	0	2152	9395	1217	2034	0	0	0	2347	9410	<.01	.71
31-35 y	1625	2223	0	158	430	2515	9953	1380	2153	0	0	0	2639	9395	1113	2040	0	0	0	1850	9410	<.01	.01
36-40 y	1754	2434	0	170	454	2641	9953	957	1924	0	0	0	775	9395	842	1990	0	0	0	108	9410	<.01	.32
41-45 y	1960	2550	0	289	448	3135	9953	1093	2303	0	0	0	772	9395	794	2020	0	0	0	0	9410	<.01	.04
Outpatient care																							
Total (18-45 y)	1370	1075	0	649	1046	1746	5404	1287	1049	0	566	974	1663	5094	1092	1049	0	410	775	1373	5208	<.01	<.01
18-19 y	—	—	—	—	—	—	—	—	—	—	—	—	—	—	—	—	—	—	—	—	—	—	—
20-26 y	1182	829	46	626	960	1469	5404	1111	909	73	544	855	1390	5094	988	996	0	411	695	1143	5208	<.01	.09
27-30 y	1351	1013	89	651	1100	1700	5404	1328	1034	0	610	1063	1692	5094	1116	1016	0	436	859	1433	5208	<.01	<.01
31-35 y	1454	1110	63	667	1167	1878	5404	1359	1106	44	572	1032	1753	5094	1171	1071	0	441	828	1572	5208	<.01	<.01
36-40 y	1380	1162	33	623	985	1728	5404	1337	1114	0	555	973	1780	5094	1065	1053	0	391	759	1280	5208	<.01	<.01
41-45 y	1386	1129	0	652	983	1788	5404	1189	967	0	555	901	1510	5094	1046	1085	0	381	700	1264	5208	<.01	.04
Pharmaceuticals[b]																							
Total (18-45 y)	268	662	0	25	72	174	3917	256	585	0	26	74	189	3419	278	750	0	16	59	164	4619	.62	.22

Table 3. All-Cause Healthcare Costs During 24-Month Follow-up, Stratified by Age, After Winsorization

	Costs (€)																					*P* Value[a]	
	Study Cohort 1							Study Cohort 2							Control Group								
	Mean	SD	Min	Q1	Med	Q3	Max	Mean	SD	Min	Q1	Med	Q3	Max	Mean	SD	Min	Q1	Med	Q3	Max	Study Cohort 1	Study Cohort 2
18-19 y	—	—	—	—	—	—	—	—	—	—	—	—	—	—	—	—	—	—	—	—	—	—	—
20-26 y	221	595	0	23	67	143	3917	208	517	0	21	54	123	3419	181	483	0	18	47	119	4619	.34	.47
27-30 y	213	569	0	23	64	138	3917	224	582	0	25	71	154	3419	271	788	0	14	55	148	4619	.14	.23
31-35 y	302	733	0	23	72	173	3917	281	634	0	26	72	185	3419	293	776	0	18	62	172	4619	.82	.74
36-40 y	288	686	0	27	71	203	3917	266	583	0	27	83	222	3419	290	764	0	16	59	172	4619	.97	.55
41-45 y	297	666	0	30	90	224	3917	279	558	0	30	97	272	3419	321	800	0	16	73	205	4619	.62	.35
Sick leave payments[c]																							
Total (18-45 y)	135	342	0	0	0	302	2074	86	253	0	0	0	169	1588	55	247	0	0	0	0	1554	<.01	<.01
18-19 y	—	—	—	—	—	—	—	—	—	—	—	—	—	—	—	—	—	—	—	—	—	—	—
20-26 y	116	288	0	0	0	302	2074	45	110	0	0	0	0	1588	37	180	0	0	0	0	1554	<.01	.46
27-30 y	135	335	0	0	0	302	2074	91	258	0	0	0	169	1588	38	208	0	0	0	0	1554	<.01	<.01
31-35 y	113	289	0	0	0	302	2074	69	212	0	0	0	169	1588	52	234	0	0	0	0	1554	<.01	.12
36-40 y	147	379	0	0	0	302	2074	96	276	0	0	0	169	1588	71	290	0	0	0	0	1554	<.01	.12
41-45 y	173	412	0	0	0	302	2074	127	337	0	0	0	169	1588	78	296	0	0	0	0	1554	<.01	.02
Aids and remedies[c]																							
Total (18-45 y)	162	249	0	0	90	202	1260	179	275	0	0	95	249	1415	186	312	0	0	89	265	1712	<.01	.34
18-19 y	—	—	—	—	—	—	—	—	—	—	—	—	—	—	—	—	—	—	—	—	—	—	—
20-26 y	118	167	0	0	69	202	1260	149	234	0	0	63	249	1415	162	252	0	0	84	265	1712	<.01	.51
27-30 y	147	234	0	0	81	202	1260	150	247	0	0	64	249	1415	155	275	0	0	50	265	1712	.59	.75
31-35 y	165	253	0	0	88	202	1260	165	256	0	0	93	249	1415	193	315	0	0	98	265	1712	.05	.06
36-40 y	169	249	0	0	95	202	1260	207	303	0	0	111	249	1415	190	340	0	0	60	265	1712	.21	.37
41-45 y	204	303	0	0	136	202	1260	226	318	0	0	152	249	1415	228	351	0	0	119	265	1712	.26	.91

Abbreviations: Max, maximum; Med, median; Min, minimum; Q1, 25th percentile; Q3, 75th percentile.

Study cohort 1: women with CIN diagnoses and a subsequent cervical conization; study cohort 2: women with CIN diagnoses and no cervical conization in the entire study period; control group: women with neither a CIN diagnosis nor a conization in the entire study period.

[a]Compared with control group with *t* test. Due to data protection regulations no analysis of age group 18-19 was possible, as patient counts were n=1-4.

[b]Including costs for outpatient pharmaceuticals, costs for inpatient applied pharmaceuticals are included in inpatient costs.

[c]Imputed costs: As information on sick leave costs as well as aids and remedies costs are not complete for all individuals in the InGef Research Database, missing sick leave and aids and remedies data was imputed by using the mean sick leave costs and aids and remedies costs of women with available sick leave data and aids and remedies data, respectively.
